# Decrease of renal resistance during hypothermic oxygenated machine perfusion is associated with early allograft function in extended criteria donation kidney transplantation

**DOI:** 10.1038/s41598-020-74839-7

**Published:** 2020-10-20

**Authors:** Franziska A. Meister, Zoltan Czigany, Katharina Rietzler, Hannah Miller, Sophie Reichelt, Wen-Jia Liu, Joerg Boecker, Marcus J. Moeller, Rene H. Tolba, Karim Hamesch, Pavel Strnad, Peter Boor, Christian Stoppe, Ulf P. Neumann, Georg Lurje

**Affiliations:** 1 Department of Surgery and Transplantation, University Hospital RWTH Aachen, Aachen, Germany; 2 Division of Nephrology, Department of Medicine II, University Hospital RWTH Aachen, Aachen, Germany; 3 Institute for Laboratory Animal Science and Experimental Surgery, University Hospital RWTH Aachen, Aachen, Germany; 4 Division of Gastroenterology and Hepatology, Department of Medicine III, University Hospital RWTH Aachen, Aachen, Germany; 5 Institute of Pathology, University Hospital RWTH Aachen, Aachen, Germany; 6 Department of Intensive Care Medicine, University Hospital RWTH Aachen, Aachen, Germany; 7 Department of Surgery, Chirugische Klinik, Campus Charité Mitte|Campus Virchow Klinikum–Charité Universitätsmedizin Berlin, Berlin, Germany

**Keywords:** Chronic kidney disease, Renal replacement therapy

## Abstract

Hypothermic oxygenated machine perfusion (HOPE) was recently tested in preclinical trials in kidney transplantation (KT). Here we investigate the effects of HOPE on extended-criteria-donation (ECD) kidney allografts (KA). Fifteen ECD-KA were submitted to 152 ± 92 min of end-ischemic HOPE and were compared to a matched group undergoing conventional-cold-storage (CCS) KT (n = 30). Primary (delayed graft function-DGF) and secondary (e.g. postoperative complications, perfusion parameters) endpoints were analyzed within 6-months follow-up. There was no difference in the development of DGF between the HOPE and CCS groups (53% vs. 33%, respectively; p = 0.197). Serum urea was lower following HOPE compared to CCS (p = 0.003), whereas the CCS group displayed lower serum creatinine and higher eGFR rates on postoperative days (POD) 7 and 14. The relative decrease of renal vascular resistance (RR) following HOPE showed a significant inverse association with serum creatinine on POD1 (r = − 0.682; p = 0.006) as well as with serum urea and eGFR. Besides, the relative RR decrease was more prominent in KA with primary function when compared to KA with DGF (p = 0.013). Here we provide clinical evidence on HOPE in ECD-KT after brain death donation. Relative RR may be a useful predictive marker for KA function. Further validation in randomized controlled trials is warranted.

**Trial registration:** clinicaltrials.gov (NCT03378817, Date of first registration: 20/12/2017).

## Introduction

Kidney transplantation (KT) has evolved as the mainstay of treatment for end-stage renal disease (ESRD)^1^. Ischemia–reperfusion injury (IRI) constitutes a significant source of morbidity and mortality after KT and is frequently associated with delayed graft function (DGF) and primary non-function (PNF)^2,3^.

In the context of a global organ shortage, extended criteria donor (ECD) kidney allografts (KA) are more and more considered for transplantation. These ECD-allografts, however, are also associated with an increased susceptibility to ischemia–reperfusion injury and a higher probability to develop PNF and/or DGF^4^. Over the last decades several strategies have been employed, aiming at reconditioning poor quality ECD-KA^5,6^. These include hypothermic- and normothermic machine perfusion (HMP and NMP), hypothermic oxygenated machine perfusion (HOPE), controlled oxygenated rewarming as well as regional perfusion protocols^7,8^. Although, numerous studies demonstrated the potential benefits of HMP in DCD and DBD (donation after brain death) kidney transplantation, the translation of this technology into routine clinical practice is still limited^9–11^.

With the increase of ECD-KT, a proper matching of donors and recipients poses a great challenge. The kidney donor profile index (KDPI) was implemented in an attempt to objectively assess KA quality and is based on a subjective macroscopic evaluation during retrieval and before KA implantation^12,13^. Real-time detection of perfusion parameters, such as flow- and renal vascular resistance (RR), for viability- and allograft quality assessment during machine perfusion are becoming increasingly important^13,14^. Although, tissue oxygen consumption is markedly decreased at 4–10 °C, there is still a significant cellular metabolism^15^. The technique of HOPE evolved in parallel to HMP and, in addition to HMP, provides active perfusate oxygenation at a partial pressure of 60–100 kPa^7,16^. In preclinical trials, end-ischemic reconditioning of KA with HOPE effectively mitigated downstream macrophage-, endothelial activation and increased cellular energy household via various mitochondrial pathways^15,17^ .

The beneficial effects of HOPE over non-oxygenated HMP and conventional cold storage (CCS) have been suggested in preclinical and retrospective clinical studies^15,18–22^. The present prospective case-matched pilot study aims to provide novel clinical evidence and compares the effects of HOPE versus CCS in human ECD-DBD kidney transplantation.

## Methods

### Trial design and ethics

**Figure 1:**
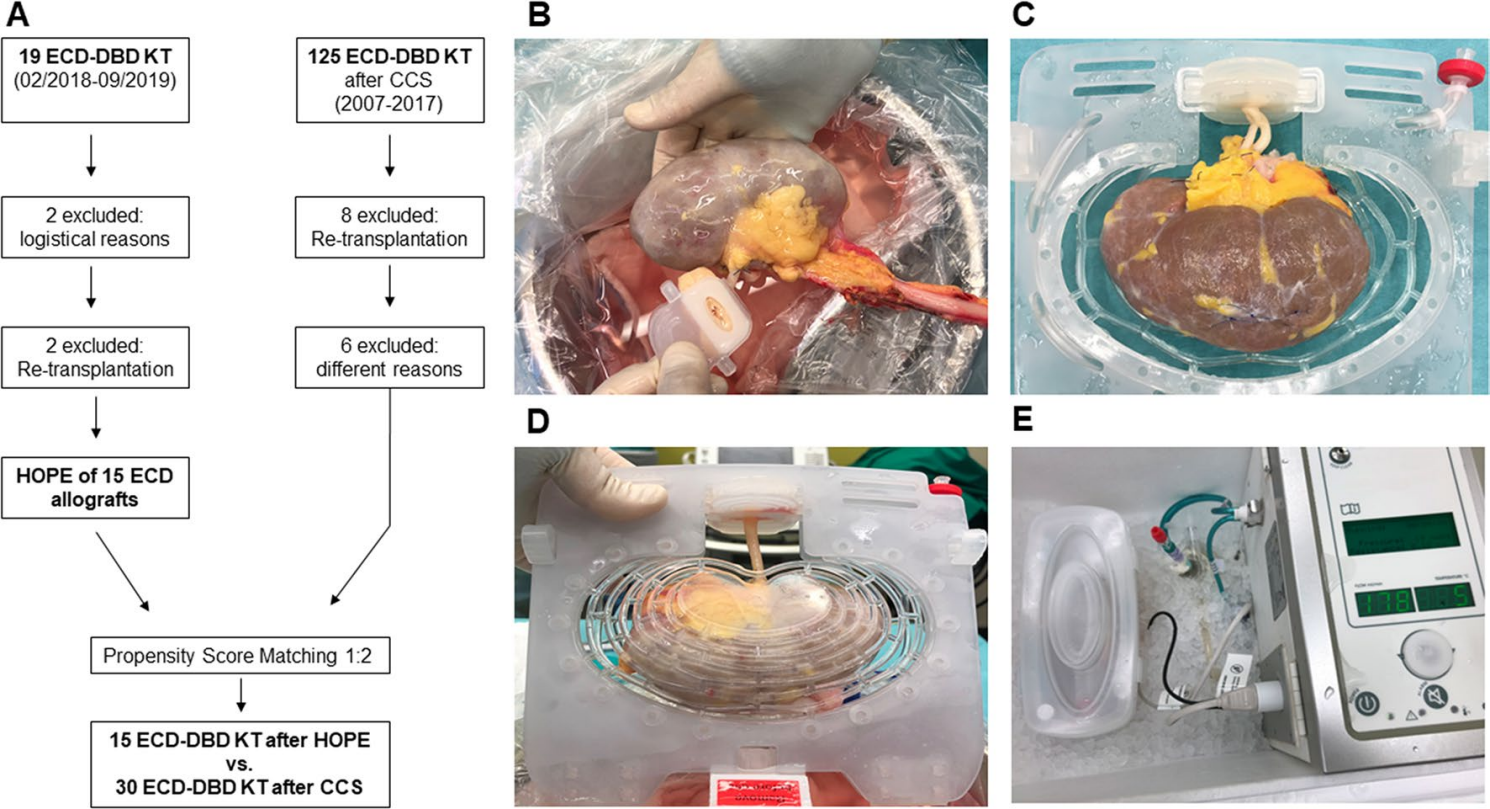
(**A**) Enrollment of hypothermic oxygenated machine perfusion (HOPE) preserved allografts, compared to a historical cohort transplanted after conventional cold storage (CCS) at the University Hospital RWTH Aachen, Aachen, Germany. (**B**) Connecting the renal artery to the renal artery cannula following back-table preparation. (**C**) Positioning of the allograft in the kidney holder mesh of the device. (**D**) The kidney holder mesh is closed. After its placement into the organ container the allograft is ready to be perfused. (**E**) Hypothermic oxygenated machine perfusion of the kidney allograft with the Kidney Transport Assist (Organ Assist b.v., Groningen, The Netherlands). *ECD*, Extended criteria donor; *DBD*, Donation after brain death, *KT*, kidney transplantation.

The present investigator-initiated prospective clinical study was conducted to evaluate the effects of HOPE on ECD kidney allografts in DBD kidney transplantation. Fifteen patients, who received ECD kidney allografts at the Department of Surgery and Transplantation, University Hospital RWTH Aachen, Aachen, Germany (UH-RWTH) between February 2018 and September 2019 were included in this study. These 15 patients were case-matched in a ratio of 1:2 to historical cohort of patients, transplanted following CCS between 2007 and 2017 (Fig. 1). The present trial was carried out in compliance with the current version of the Declaration of Helsinki, good clinical practice guidelines (ICH-GCP) as well as all national legal and regulatory requirements. The institutional review board of the University RWTH Aachen has approved the study protocol, including consent form and patient information leaflet (EK 184/17). Members of the study team have completed a course in good clinical practice as certified by the German Medical Chamber. The protocol of the trial was registered at clinicaltrial.gov (NCT03378817; Date of first registration: 20/12/2017) and an a priori study protocol was published^23^.

### Extended criteria donor and organ procurement

Extended criteria donor (ECD) kidney allografts were retrieved by local procurement teams within the Euro Transplant network. Following cross-clamping, allografts were removed and transported in a standardized fashion on crushed ice. Required data regarding the donor and the allograft were collected in a standardized manner and were transferred to designated case report files (CRFs). As per previous studies, ECD was defined as deceased donors ≥ 60 years old^24^. Kidney allografts from donors aged between 50 and 59 years old were considered as ECD if they fulfilled at least two of the following conditions: (1) cerebrovascular cause of death; (2) history of arterial hypertension; or (3) serum creatinine greater than 1.5 mg/dL (132.6 µmol/L) at the time of collection^25^. The classification of the ECD allografts was based exclusively on the above-mentioned clinical criteria and no preallocation biopsy was performed to determine allograft quality.

### Study participants and data collection

German or English-speaking patients older than 18 years, suffering from end-stage-renal-disease, listed for KT, and receiving ECD-KA at the University hospital RWTH Aachen were included. Patients undergoing re-KT, pregnant patients, patients undergoing combined- or living donor transplantation procedures, receiving any investigational drug within 30 days before inclusion or unable to provide an informed consent were excluded (4 exclusions in total). Written informed consent from the participants was collected in our out-patient clinic. Data were obtained using study-specific CRFs completed by the members of the study team.

### Technical details

End-ischemic HOPE (Kidney Transport Assist; Organ Assist b.v., Groningen, The Netherlands) was applied in the operating theater of the UH-RWTH after regular organ procurement, transport and back-table preparation via the renal artery for at least 60 min with a perfusion pressure of 25 mmHg using 1 L of recirculated perfusate (Belzer machine perfusion solution, Bridge to Life, London, UK) at 0–4 °C, (Fig. 1). The perfusion settings were chosen according to previous clinical studies with non-oxygenated HMP and based on available preclinical experiences with HOPE^15,26,27^. Oxygenation of the perfusate was carried out with medical-grade O_2_ with a pO_2_ of 60–80 kPa by an oxygenator included as a disposable part of the setup. Perfusion parameters were automatically registered by the device. Grafts were flushed with Belzer machine perfusion solution to wash out the residual histidine-tryptophan-ketoglutarate (HTK) solution, immediately before HOPE. Storage, management, and use of the aforementioned medical products were carried out according to the manufacturer’s guidelines.

Aside from performing HOPE of the KA in the intervention group, all procedures including peri-, intra- and postoperative management were identical in the HOPE and CCS groups. Surgical procedures were performed in a standardized fashion according to our institutional protocols. Briefly, KA were implanted heterotopically into the iliac fossa. The renal vein was anastomosed end-to-side to the external iliac vein. Renal artery anastomosis to the external iliac artery was performed in the same fashion. Following reperfusion of the KA, the ureter was implanted into the bladder. Anti-reflux reconstruction has been performed according to Lich-Gregoir and the uretero-cystostomy was stented^20^ with a 6-French double-J stent^28,29^.

The applied immunosuppressive regimen was based on intraoperative induction therapy with intravenous methylprednisolone and either basiliximab or thymoglobulin. Postoperatively, oral doses of prednisolone, tacrolimus and mycophenolate mofetil were administered in a standardized fashion as previously described^20^.

### Study endpoints and statistical analysis

The primary endpoint of the study was delayed graft function (DGF), defined as the need for dialysis within the first 7-days post-KT. Secondary endpoints included creatinine reduction ratio day 2 (CRR2 = creatinine day 1 − creatinine day 2/creatinine day 1) and CRR5 (CRR5 = pre-transplant creatinine − creatinine day 5/pre-transplant creatinine), alterations of renal resistance- and flow during HOPE, the incidence of postoperative complications within the first 90 postoperative-days as assessed by the Clavien-Dindo classification (CD) and the comprehensive complication index (CCI)^30^, duration of intensive care- and hospital stay, 6 months recipient-, graft survival, death censored graft survival^31^ and renal function (assessed by serum creatinine and estimated glomerular filtration rate—eGFR) at 3- and 6 months post-transplantation. Perfusion parameters including flow and renal vascular resistance (RR) were analyzed as absolute values of RR (and flow) as well as relative alterations during HOPE compared to baseline.

Propensity score matching was used for patients with HOPE-treated KA and for those undergoing CCS^32^. Matching criteria included urinary output over the last 24 h before retrieval, cold ischemic time (CIT), and the recipients' Charlson comorbidity index (CCi) before KT^33^. Continuous variables were compared using the Mann Whitney U test or one-way analysis of variance (ANOVA). Two-way analysis of variance (ANOVA) was used for the time-course analysis of laboratory parameters. Spearman's rank-order was used to further analyze the association of various clinical and perfusion parameters. Data were expressed as mean and standard deviation. Statistical significance was defined as p < 0.05 and the statistical analysis has been performed using SPSS Statistics v24 (IBM Corp., Armonk, NY, USA).

## Results

### Patient follow up

No participant who received a HOPE-treated kidney allograft, was lost to follow-up. One recipient was readmitted and died 5 months following KT due to a fulminant influenza virus infection with a well-functioning KA.

### Baseline recipient and donor characteristics

Fifteen patients (9 women and 6 men; mean age 60 ± 9 years) transplanted after HOPE-treatment of the KA were compared to a historical cohort of 30 patients (11 women and 19 men; mean age 60 ± 10 years) transplanted after CCS between 2007 and 2017 (1:2 matching). Age and body mass index of both donor and recipient, as well as CIT, warm ischemia time (WIT) and recipient comorbidities assessed by the Charlson comorbidity index (CCi), were equally distributed in both groups without statistical differences. Recipient- and donor characteristics are summarized in Table 1.

**Table 1:** Donor-, recipient- and allograft characteristics.

	Recipient		p-value
	HOPE (N = 15)	CCS (N = 30)	
Age	60 ± 9	60 ± 10	0.809
BMI	26 ± 6	27 ± 6	0.386
Sex ratio (female:male)	7:8 (47%:53%)	11:19 (36%:64%)	0.519
CCi^a^	4.7 ± 1.8	4.7 ± 1.6	0.980
EPTS^b^	60 ± 19	58 ± 20	0.736
Etiology of ESRD	ADPKD: 7 (47%) Diabetic KD: 1 (7%) Hypertensive KD: 0 (0%) Nephritis: 5 (33%) Other: 2 (13%)	ADPKD: 4 (13%) Diabetic KD: 2 (7%) Hypertensive KD: 4 (13%) Nephritis: 11 (37%) Other: 9 (30%)	0.103

	Donor		p-value
	HOPE (N = 15)	CCS (N = 30)	
Age	66 ± 12	66 ± 8	0.847
BMI	28 ± 7	27 ± 4	0.981
Sex ratio (female:male)	9:6 (60%:40%)	16:14 (53%:47%)	0.671
Cause of death	CVA: 9 (60%) Anoxia: 5 (33%) Trauma: 1 (7%)	CVA: 19 (64%) Anoxia: 10 (33%) Trauma: 1 (3%)	0.941
Diabetes mellitus	Yes: 8 (53%) No: 6 (40%) Unknown: 1 (7%)	Yes: 10 (33%) No: 12 (40%) Unknown: 8 (27%)	0.233
Arterial hypertension	Yes: 8 (53%) No: 5 (33%) Unknown: 2 (14%)	Yes: 17 (57%) No: 5 (16%) Unknown: 8 (27%)	0.355
Diuresis last 24 h (ml)	2 550 ± 1 800	3 180 ± 2 300	0.224
KDPI	87 ± 14	87 ± 18	0.708
Serum crea (mg/dl)	1.9 ± 1.4	1.4 ± 0.7	0.457
CIT (min)	646 ± 227	674 ± 214	0.563
WIT (min)	36 ± 9	36 ± 11	0.843

Values are given as mean ± standard deviation or numbers and (percent).

*HOPE*, hypothermic oxygenated machine perfusion; *CCS*, conventional cold storage; *BMI*, body mass index; *CCi,* charlson comorbidity index; *KDPI,* Kidney donor profil index; EPTS, estimated post-transplant survival; *ESRD,* end-stage renal disease; CIT, cold ischemia time; WIT, warm ischemia time.

^a^According to Charlson et. al.^33^.

^b^According to Organ Procurement and Transplantation Network^45^.

### Kidney allograft function

Delayed graft function did not differ significantly between the groups. Eight patients in the HOPE group and 10 patients in CCS suffered from DGF (53% vs. 33% p = 0.197). While there was no PNF in the CCS group, one patient receiving a HOPE-treated KA (donor age: 50 years, donor BMI: 46, total preservation time: 923 min) developed PNF.

### Renal retention parameter

**Figure 2:**
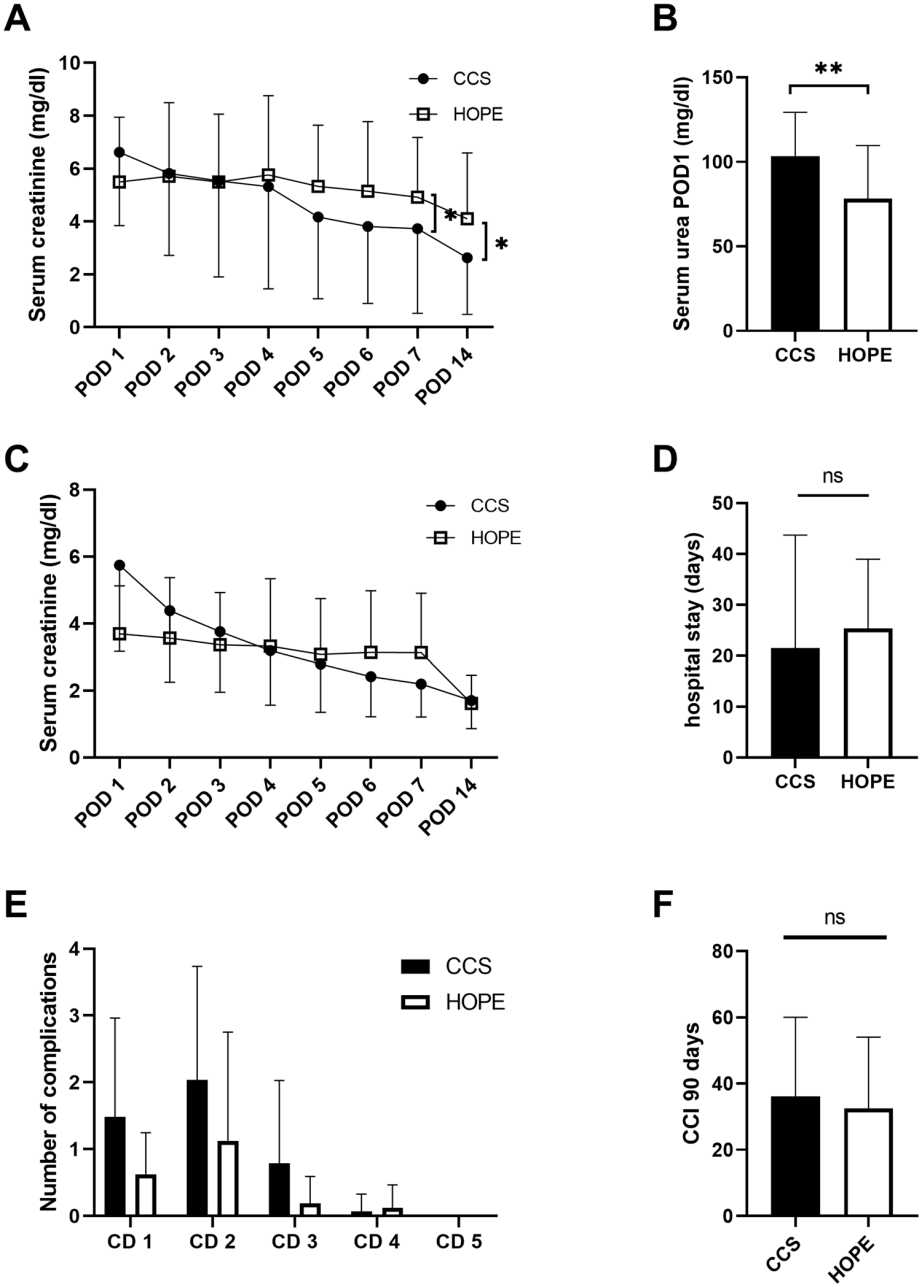
(**A**) Time course of serum creatinine levels during the early postoperative phase in the hypothermic oxygenated machine perfusion (HOPE) and conventional cold storage (CCS) groups (n = 45). Serum creatinine was higher after HOPE at postoperative day (POD) 7 (4.9 ± 2.2 mg/dl vs. 3.7 ± 3.2/ mg/dl p = 0.033) and POD 14 (4.1 ± 2.5 mg/dl vs. 2.6 ± 2.1 mg/dl p = 0.034). (**B**) Serum urea levels on POD1 were higher in CCS than in the HOPE group (78 ± 32 mg/dl vs. 103 ± 26 mg/dl p = 0.003; MWU). (**C**) Time course of serum creatinine levels in allografts with PF during the early postoperative days in HOPE and CCS groups (n = 24; MWU). Allografts with DGF were excluded, considering the effect of dialysis on these values. (**D**) Duration of hospital stay following kidney transplantation in HOPE and CCS groups (25 ± 13d vs. 21 ± 22d p = 0.570; MWU). (**E**) Postoperative 90-day complications assessed by the Clavien-Dindo (CD) classification following HOPE and CCS, without significant between-group differences. (**F**) Postoperative 90-day complications assessed by comprehensive complication index in HOPE and CCS groups (32 ± 22 vs. 36 ± 24 p = 0.647; MWU). *p < 0.05, **p < 0.01.

Laboratory parameters over the first postoperative days, including serum creatinine, serum urea and eGFR were comparable in both groups (Table 2). While serum urea was lower in the HOPE group on POD 1 (78 ± 32 mg/dl vs. 103 ± 26 mg/dl; p = 0.003) (Fig. 2B), serum creatinine was significantly lower in the CCS group on POD 7 (4.9 ± 2.2 mg/dl vs. 3.7 ± 3.2 mg/dl; p = 0.033) and POD 14 (4.1 ± 2.5 mg/dl vs. 2.6 ± 2.1 mg/dl; p = 0.034) (Fig. 2A). Likewise, eGFR was higher in CCS patients on POD 7 (35 ± 42 vs. 14 ± 10 ml/min; p = 0.027). There was no difference in CRR2 and CRR5 levels between the HOPE and CCS groups. (Table 2).

**Table 2:** Clinical outcome in terms of primary- and secondary endpoints.

	HOPE (N = 15)	CCS (N = 30)	p-value
DGF	8 (53%)	10 (33%)	0.197
PNF	1 (7%)	0 (0%)	0.333
6-months graft survival (%)	87	100	0.041
6-months DC graft survival (%)	93	100	0.333
ICU stay (days)	3 ± 3	3 ± 4	0.600
CRR 2	− 0.5 ± 0.32	0.13 ± 0.27	0.064
CRR 5	0.11 ± 0.37	0.32 ± 0.26	0.071
eGFR (ml/min) POD 1	11 ± 6	9 ± 4	0.341
eGFR (ml/min) POD 3	13 ± 11	16 ± 13	0.401
eGFR (ml/min) POD 5	13 ± 12	22 ± 16	0.051
eGFR (ml/min) POD 7	14 ± 10	31 ± 15	0.023
eGFR (ml/min) POD 14	22 ± 19	35 ± 18	0.051
eGFR (ml/min) 3 months	29 ± 16	35 ± 19	0.300
eGFR (ml/min) 6 months	32 ± 14	38 ± 17	0.276
Serum Crea (mg/dl) 3 months	2.6 ± 1.4	2.1 ± 1.1	0.253
Serum Crea (mg/dl) 6 months	2.6 ± 1	2.1 ± 1.3	0.352

Values are given as mean ± standard deviation or numbers and (percent).

*HOPE*, hypothermic oxygenated machine perfusion; *CCS*, conventional cold storage; *DGF,* delayed graft function; *PNF,* primary non-function; *DC*, death censored; *ICU,* intensive care unit; *Crea*, creatinine *CRR*, creatinine reduction ratio; *eGFR,* estimated glomerular filtration rate; *POD,* postoperative day.

Considering the potential effects of hemodialysis on these laboratory parameters, patients with DGF were excluded from further analysis and the aforementioned differences disappeared (Fig. 2C).

### Perfusion parameters

**Figure 3:**
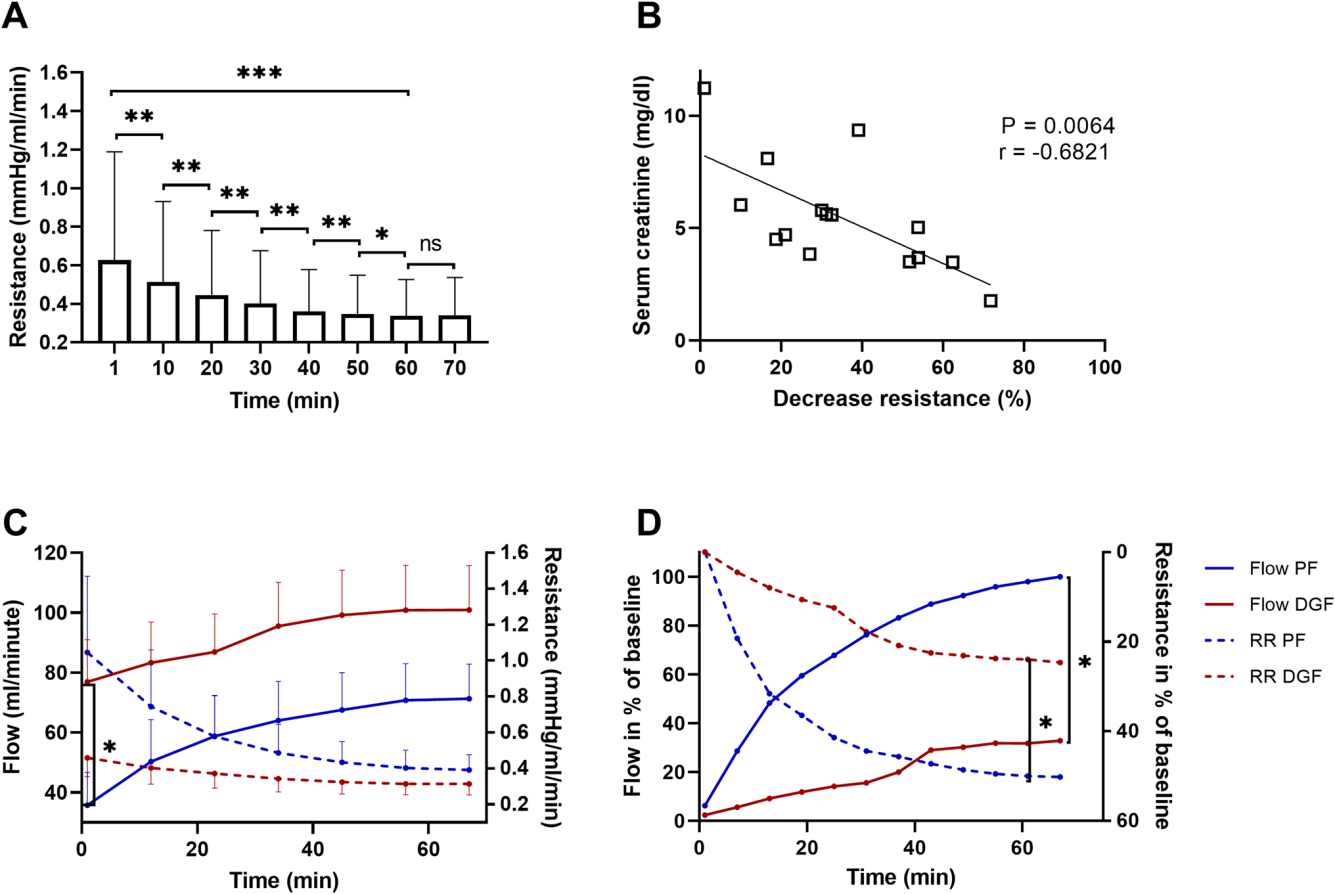
(**A**) Decrease of renal resistance (RR) during hypothermic oxygenated machine perfusion (HOPE). During the first hour of HOPE, RR changes significantly and reaches a plateau in the later phase of perfusion (Wilcoxon rank sum test). (**B**) Alterations of relative RR correlate with recipients’ serum creatinine on POD1 (Spearman's rank-order). (**C**) Development of flow and RR in kidney allografts with primary function (PF, blue) and delayed graft function (DGF, red) during HOPE. Initial flow was lower in the PF group (36 ± 22 ml/min vs. 77 ± 45 ml/min p = 0.040; MWU). (**D**) Increase of mean flow and decrease of mean RR compared to baseline during HOPE in PF and DGF kidney allografts. Mean alterations of flow and RR were higher in the PF than in the DGF group (RR: 54 ± 16% vs. 25 ± 15% p = 0.013; MWU) *p < 0.05, **p < 0.01 ***p < 0.001.

The mean time on HOPE was 152 ± 92 min (range: 70–331 min). The predefined minimum of 60 min of perfusion was extended while awaiting crossmatch results when preoperative dialysis of the recipient was necessary or for other logistic reasons. The total cold preservation time was kept as short as possible (646 ± 227 min) and organ preservation was not prolonged due to HOPE logistics. Mean renal artery flow was 63 ± 18 ml/min at start of HOPE and increased to 87 ± 46 ml/min after 60 min. The maximal flow was measured immediately before disconnecting the kidney from the machine with a mean of 90 ± 47 ml/min (Fig. 3C).

During HOPE, a decrease in RR was accompanied by a parallel increase in renal flow, reaching a plateau after 60 min. There were no significant differences in RR (and in flow) during the later phase of KA perfusion (Fig. 3A).

A significant inverse association was found between the relative decrease of RR during HOPE with serum creatinine on POD1 and 2 (r = − 0.682, p = 0.006; r = − 0.612, p = 0.015, respectively) (Fig. 3B), serum urea on POD 4, 5, 6, and 14 (r = − 0.676, p = 0.008; r = − 0.594 p = 0.032; r = − 0.631, p = 0.021; r = − 0.707; p = 0.010, respectively) and eGFR on POD 1 and 2 (r = 0.663, p = 0.007; r = 0.538, p = 0.039, respectively).

More importantly, the relative RR values following 60 min of HOPE were significantly higher in KA with primary function compared to those developing DGF (54 ± 16% vs. 25 ± 15%; p = 0.013, respectively; see Fig. 3D).

### Postoperative surgical complications and length of hospital stay

Postoperative complications as assessed by the Clavien-Dindo classification (CD1: 10 vs. 43 p = 0.141; CD2: 18 vs. 59 p = 0.111; CD3: 3 vs. 23 p = 0.119; CD4: 2 vs. 2 p = 0.464) and the CCI (32 ± 22 vs. 36 ± 24 p = 0.647) were lower in the HOPE group but did not reach statistical significance (Fig. 2E,F). In line with the findings above, neither ICU stay (3 ± 3 days vs. 3 ± 4 days; p = 0.600) nor hospital stay (25 ± 13 days vs. 21 ± 22 days; p = 0.570) differed significantly between the CCS and HOPE groups, respectively (Table 2, Fig. 2D).

### Six-months allograft survival

During the defined follow-up period of 6 months, one patient in the HOPE group deceased due to influenza virus infection with a well-functioning graft and one patient developed PNF. In our case-matched cohort of patients receiving ECD allografts following CCS, no recipient has died over the observation period. Graft survival after 6 months showed significant differences with 87% in the HOPE group and 100% in the CCS group (p = 0.041). No significant difference was observed in death censored graft survival (93% vs. 100%; p = 0.333). Retention parameter 6-months after the transplantation were similar in both groups. Detailed clinical data are presented in Table 2.

## Discussion

The present study demonstrates the safety and feasibility of end-ischemic HOPE in human ECD kidney transplantation. While our primary endpoint—i.e. the difference in the number of transplanted patients with delayed graft function—was not reached, we show unique clinical data on the effects of HOPE in KT using ECD-DBD allografts. In this setting, a relative decrease of renal resistance during HOPE was identified as a potential parameter to better predict post-transplant kidney allograft function.

Kidney transplantation evolved as the mainstay of treatment for ESRD. Donor allografts that would have previously been deemed unsuitable for transplantation due to their compromised quality, have nowadays become an essential part of the organ pool and are being routinely transplanted in ECD programs^7,34^. As the susceptibility to ischemia–reperfusion injury and the risk of DGF and PNF are considerably higher in ECD allografts, pretransplant viability assessment and reconditioning of these KA is of utmost clinical importance^35^. The present prospective clinical trial is one of the first controlled clinical studies reporting the use of HOPE as an innovative end-ischemic machine perfusion approach in human KT using ECD-DBD kidney allografts.

Although, pre-clinical and small clinical case-series suggested the potential role of perfusion parameters (e.g. RR and arterial flow) in the quality assessment of KA before transplantation^35,36^, only very limited data is available on the value of these parameters in the setting of end-ischemic HOPE and ECD-DBD kidney transplantation^13,37^. In non-oxygenated HMP lower absolute renal vascular resistance values were generally associated with better post-transplant outcome and overall KA function^14^. In the present study, however, we could not confirm these findings as the initial RR in KA with good primary function was significantly higher compared to the RR in those who developed DGF^13,38^. To further explore these findings, we investigated the relative changes of RR and flow parameters during HOPE. One of the most important observations was a significant association between laboratory parameters of kidney function and the relative decrease of RR during the first 60 min of HOPE followed by a consequential increase in flow. These results are in line with Bissolati et. al. describing the assessment of dynamic RR patterns in a non-oxygenated HMP setting as being more predictive of post-KT outcome compared to the traditional Karpinski’s histological analysis of pre-transplant biopsies^13^.

Jochmans et. al. validated the association between RR and kidney allograft quality. In their study, the authors analyzed RR of 302 KA treated by non-oxygenated HMP after DCD or DBD and identified the absolute RR value at the end of the perfusion to be an independent risk factor for DGF and 1-year graft failure^14^. Despite that, the authors do not recommend the use of the absolute RR as a stand-alone quality assessment tool. This recommendation is supported by a study by Sonnenday et. al. where 11 out of 14 ECD-KA were successfully transplanted after they were discarded by multiple centers due to poor perfusion parameters^39^. In the present HOPE-study, no KA was rejected solely based on perfusion parameters.

With regards to our findings, we assume that relative alterations of RR during HOPE are more important than the absolute baseline value and that a relative RR decrease may serve as a quality indicator for post-transplantation KA function in ECD-DBD kidney transplantation. Interestingly, after 1 h of HOPE perfusion, RR and flow parameters have reached their plateau and we could not detect any further significant alterations (Fig. 3). Based on this observation, the early variation of RR may be a suitable real-time clinical parameter to determine how long HOPE perfusion should be performed to achieve a desired reconditioning effect.

In contrast to the results of recent cohort studies on NMP and HMP^11,27,40,41^, the present trial did not show significant differences in the development of DGF in KA undergoing HOPE treatment compared to KA treated with CCS (p = 0.197). Similarly, a recent report by Summers et. al. could not show a clinical benefit of non-oxygenated HMP in kidney transplantation. The authors used cold pulsatile machine perfusion in a British cohort of 51 KA after DCD and transplanted the kidney pair of the same donor after CCS. They could neither show significant differences in the incidence of DGF nor in the incidence of acute cellular rejection^42^.

The findings of the present study should be interpreted in light of potential limitations. First, the most important and obvious limitation of our study is the single-center, non-randomized pilot study design which resulted in a limited power of our statistical analysis when interpreting between-group differences in terms of clinical outcomes such as DGF and complications. Therefore, no significant differences have been found in terms of any of the clinically relevant primary and secondary outcome measures. These non-superior results limit the value of any definitive conclusion of our study about the use of HOPE as an alternative method to CCS. Second, we acknowledge that DGF may be an inadequate primary endpoint, especially in case-matched study design. Thresholds for dialysis initiation can differ between clinicans and may have changed over the years^43,44^. This circumstance may have led to a higher rate of dialysis compared to our historical cohort. Clinical outcomes, such as complications, DGF, and death censored graft survival were not superior in the HOPE group compared to CCS. While serum urea showed significantly favorable alterations in the HOPE group on POD 1, it was superior in CCS during the subsequent days.

In conclusion, the present study provides novel clinical data on the use of HOPE in ECD-DBD kidney transplantation. The relative decrease of renal vascular resistance as a novel marker to better predict post-transplant allograft function may be an important parameter for real-time viability assessment of HOPE-treated kidney allografts. Further validation in randomized controlled clinical trials is warranted.

## Data Availability

All relevant data has been reported within the manuscript. Further supplementary datasets can be obtained upon written request addressed to the corresponding author.

## Abbreviations

CCI: Comprehensive complication index
CCi: Charlson comorbidity index
CCS: Conventional cold storage
CRR: Creatinine reduction ratio
BMI: Body mass index
DBD: Donation after brain death
DCD: Donation after cardiac death
DGF: Delayed graft function
ECD: Extended criteria donor
EPTS: Estimated post transplantation survival
eGFR: Estimated glomerular filtration rate
HOPE: Hypothermic oxygenated machine perfusion
HMP: Hypothermic machine perfusion
HTK: Histidine-tryptophan-ketoglutarate solution
KA: Kidney allografts
KDPI: Kidney donor profile index
KT: Kidney transplantation
MWU: Mann–Whitney-U test
PF: Primary function
PNF: Primary graft non-function
POD: Postoperative day
RCT: Randomized controlled trial
RR: Renal resistance
